# Genetic variation of *Borreliella burgdorferi* in Fairfax County, Virginia, targeting the OspC gene in white-footed mice

**DOI:** 10.3389/fmicb.2022.998365

**Published:** 2022-11-18

**Authors:** Sonya G. Zawada, Michael E. von Fricken, Thomas A. Weppelmann, Masoumeh Sikaroodi, Patrick M. Gillevet

**Affiliations:** ^1^Division of Science and Math, NorthWest Arkansas Community College, Bentonville, AR, United States; ^2^Department of Biology, George Mason University, Fairfax, VA, United States; ^3^Department of Global and Community Health, George Mason University, Fairfax, VA, United States; ^4^Morsani College of Medicine, University of South Florida, Tampa, FL, United States

**Keywords:** Lyme disease, borreliosis, OspC gene, genetic variability, white-footed mouse, *Peromyscus leucopus*, *Borrelia burgdorferi* spirochete, *Borreliella*

## Abstract

Outer surface protein C (OspC) is a commonly used marker in population studies of *Borreliella* to differentiate types and establish evolution over time. Investigating the *ospC* genetic types of *Borreliella burgdorferi* across multiple organ tissues of white-footed mice has the potential to contribute to our understanding of Lyme disease and the wide spectrum of clinical presentation associated with infection. In this study, five unique tissue types were sampled from 90 mice and screened for *B. burgdorferi* infections. This initial screening revealed a 63% overall *B. burgdorferi* infection rate in the mice collected (57/90). A total of 163 tissues (30.4%) tested positive for *B. burgdorferi* infections and when mapped to *Borreliella* types, 143,894 of the initial 322,480 reads mapped to 10 of the reference sequences in the *ospC* strain library constructed for this study at a 97% MOI. Two tissue types, the ear and the tongue, each accounted for 90% of the observed *Borreliella* sequence diversity in the tissue samples surveyed. The largest amount of variation was observed in an individual ear tissue sample with six *ospC* sequence types, which is equivalent to 60% of the observed variation seen across all tested specimens, with statistically significant associations observed between tissue type and detected *Borreliella*. There is strong evidence for genetic variability in *B. burgdorferi* within local white-footed mouse populations and even within individual hosts by tissue type. These findings may shed light on drivers of infection sequalae in specific tissues in humans and highlights the need for expanded surveillance on the epigenetics of *B. burgdorferi* across reservoirs, ticks, and infected patients.

## Introduction

White-footed mice (*Peromyscus leucopus*) are the principle reservoir for *Borreliella burgdorferi* (Spirochaetales: Spirochaetaceae) in the eastern United States (Peavey and Lane, 1995), with their habitat ranging from Maine to Alabama and stretching as far west as Arizona (Lackey et al., 1985). Transmitted by *Ixodes* (Acari: Ixodidae) species ticks (Burgdorfer et al., 1982; Balashov et al., 1997), *B. burgdorferi* resides in the tick midgut, eventually migrating to tick salivary glands and infecting the host after a period of attachment (Zung et al., 1989; Peavey and Lane, 1995; Ogden et al., 2011). The bacterium replicates and passes through tick tissues eventually reaching the salivary glands and inoculating the dermis of the mammalian host. It is during this migration that changes in the expression of the outer surface proteins of the *Borreliella* spirochete occur. Prior to taking a bloodmeal, the *Borreliella* spirochete maintains outer surface protein A (OspA). However, once the tick initiates feeding, the dominant outer surface protein expression switches from OspA to OspC. OspC is required for productive mammalian infection to occur, with peak expression during tick-feeding at the 48-h mark (Schwan and Piesman, 2002). In previous studies, *B. burgdorferi* spirochetes with mutated OspC were not detected in mouse tissue culture post exposure, and the inflammatory response seen in human hosts is not detectable in the mouse reservoir (Lin et al., 2020; Coburn et al., 2021). The *ospC* marker has since become a commonly used gene target in population studies of *B. burgdorferi* to differentiate types and establish variability of the spirochete.

Recent research into the genetics of *Borrelia* have resulted in the splitting of the genus into two genera, *Borrelia* and *Borreliella* (Adeolu and Gupta, 2014; Gupta, 2019). *Borreliella burgdorferi* (previously categorized as *Borrelia burgdorferi*) infections are commonly reported from the United States, Europe, and Asia. The strains responsible for Lyme disease infections in Europe (*B. garinii*, *B. afzelii,* and *B. burgdorferi*) are primarily associated with neurological and dermal clinical manifestations respectively, while infections in the United States, originating from the *B. burgdorferi sensu stricto* strains, are most associated with skin lesions and arthritis (Heymann and Ellis, 2012). These differences in clinical presentation are thought to be related to variance in their dissemination and inflammatory potential (Dykhuizen et al., 2008; Cerar et al., 2016).

Evidence has shown a link between *Borreliella* strains and clinical manifestations in mice and humans (Seinost et al., 1999; Wang et al., 2001, 2002; Earnhart et al., 2005; Lagal et al., 2006; Dykhuizen et al., 2008; Hanincova et al., 2013). Investigating the genetic diversity of *B. burgdorferi,* specifically targeting *ospC* across multiple organ tissues of white-footed mice, has the potential to contribute to our understanding of Lyme disease and the wide spectrum of clinical presentation associated with infection.

## Materials and methods

### Field collections

Mice were collected in Fairfax County, Virginia utilizing Sherman® live steel traps in spring/summer/fall seasons and museum special traps in winter, as previously described (Zawada et al., 2020). Between forty and fifty traps were set along brush piles bordering fields at dusk with a bait mixture of peanut butter and oatmeal, marked with flagging tape, and GPS-tagged. Collected mice were documented, given a field identification number, wrapped in a piece of newspaper, and brought back to the laboratory for tissue harvest and sample processing. Specimens collected *via* Sherman® live steel traps were dispatched in a CO_2_ chamber and those collected *via* museum special traps were dispatched *via* vertebral dislocation upon triggering the trap mechanism.

Geographic localities for trapping sites are as follows: I-66 Transfer Station (38.851443–77.380081), Goodwood (38.834570–77.358750), Huntley Meadows Park (38.756417–77.115347), Graves (38.771210–77.095720), Stoneybrooke (38.770943–77.096315).

### Laboratory processing

Collected mice were necropsied to separate spleen, liver, ear, tongue, tail, heart, and kidney tissues prior to storage in separate sterile microcentrifuge tubes at −80°C. DNA extraction was performed using the Qiagen DNeasy Blood & Tissue Kit according to the manufacturer’s instructions, diluted (1: 5 in DEPC water), and both the original DNA and dilutions were stored at −80°C for future use.

Detection of *B. burgdorferi ospC* was performed on all mouse tissues using a semi-nested PCR protocol with the first round amplifying a 597 base pair fragment and the second amplifying a 314 base pair fragment. The first PCR was performed using extracted DNA as a template and a combination of two forward primers (WangEF) and (LinF) with a reverse primer (LinR). The second PCR was performed using template DNA from the first PCR, a M13 tagged forward primer (WangIF M13F), and a M13 reverse tagged primer (WangER M13R) found in (Wang et al., 1999) using the Qiagen DNeasy Blood & Tissue Kit. Primer sequences can be found in Table 1.

**Table 1:** PCR primers and sequences.

Primer	Sequence
WangEF	AAAGAATACATTAAGTGCGATATT
WangER	GGGCTTGTAAGCTCTTTAACTG
WangIF	TTGTTAGCAGGAGCTTATGCAATATC
Lin ospC4R	TTTTTTGGACTTTCTGCCACA
Lin ospC3F	AAGTGCAGATATTAATGACTTTA

PCR products from the second inner reaction containing the M13 sequences were put through a final PCR to incorporate a series of specific adapters and tags that attached specifically to the M13 sequences. PCR products from all samples were pooled together based on the strength of the band visualized on a 1% agarose gel. The pooled samples were purified prior to sequencing using Agencourt AMPure XP (Beckman Coulter, Inc.). Nextgen sequencing was performed using the Ion Torrent Personal Genome Machine.

### Genetic analysis

The results obtained from the sequencing yielded 322,480 reads. The bioinformatics program Geneious, version 10.0, was used to analyze sequence data *via De Novo* assembly, to conduct multiple alignments, develop a reference library, BLAST search, construct phylogenetic trees, and for reference mapping. The MBAC Galaxy Portal was then used to organize and rename sequence reads and with the program FigTree to customize phylogenetic trees. Several in-house Perl scripts were also used to create tables, sort reads, and rename sequences.

The 322,480 fasta read master files were put through a *De Novo* assembly in Geneious and the resulting contigs were then uploaded into BLAST. The 20 unique strain reference matches from the BLAST search were recorded by accession number along with 27 additional strain references from BorreliaBase^1^, an online database that tracks and stores genetic information related to *Borrelia* spp. and different strains found worldwide. These matched references were imported into Geneious to build a reference library of 47 sequences. The strain references in the library were aligned to each other and trimmed to the *ospC* primers that were utilized for the PCR. References that were within 3% similarity to each other were removed from the library to eliminate redundancy, after which a neighbor-joining tree was built in Geneious to examine phylogenetic distance. The final number of reference strain sequences remaining in the library was reduced to 30 unique sequences and used for the remainder of the analysis.

### Mapping to references and inclusion criteria

The 322,480 sample reads were mapped to the reference library, of which, 143,894 reads had a 97% minimum overlap identity (MOI) resulting in the production of 10 contigs that corresponded to 10 unique references from the reference library. The remaining unused reads were further examined and mapped back to the reference library at a lower MOI to determine if novel variations were being lost or if these unused reads contained errors that precluded them from being incorporated into the original 97% MOI mapping.

### Statistical analysis

All data were analyzed and cleaned using STATA v. 14.1 (StataCorp. 2015. College Station, TX). Categorical data were coded in a binary fashion and analyzed using a simple logistic regression to determine the likelihood of other tissues testing positive, when compared to positive samples within the same mouse as well as the association between the number of detected types by mouse to determine if specific tissues were prone to more genetic diversity. An Analysis of Variance (ANOVA) was attempted, but the variance was not equal between groups, and thus an inappropriate test for this dataset. Z-scores and 95% confidence intervals for odds ratios were calculated to capture magnitude of association with a *p*-value of <0.05 considered significant.

### Ethics statement

An IACUC was completed and approved by George Mason University under the Institutional Biosafety Committee (IBC) protocol# 12-28Mod1. Trapping was conducted under the Virginia Department of Game and Inland Fisheries (VADGIF) permit number 032199, 035522, 046610, and 050364.

## Results

### *Borreliella burgdorferi* types

**Figure 1:**
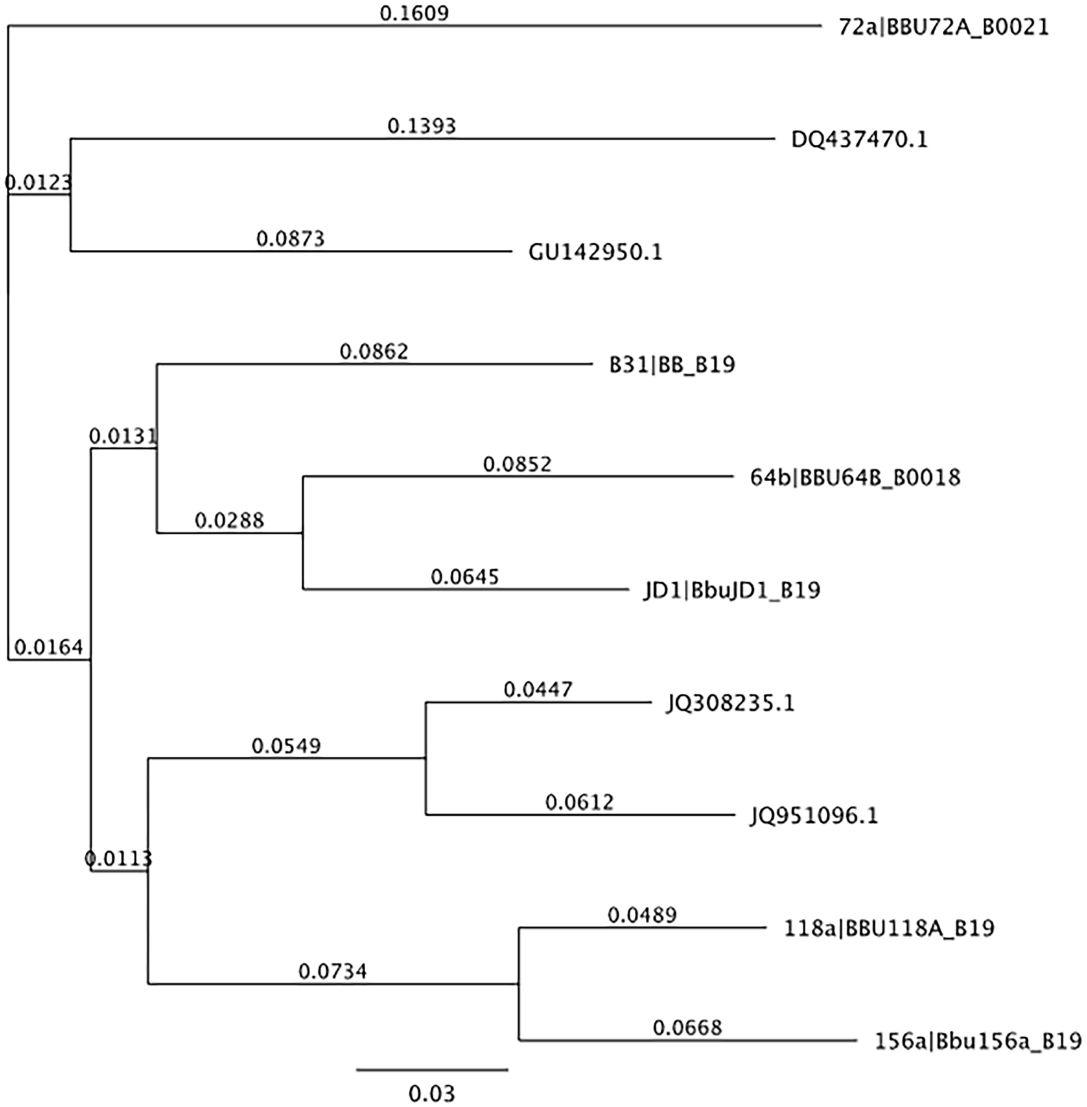
Neighbor-joining tree of OspC reference library. Neighbor-joining tree of OspC reference library created using Geneious version 10.0 created by Biomatters. Available from http://www.geneious.com.

**Figure 2:**
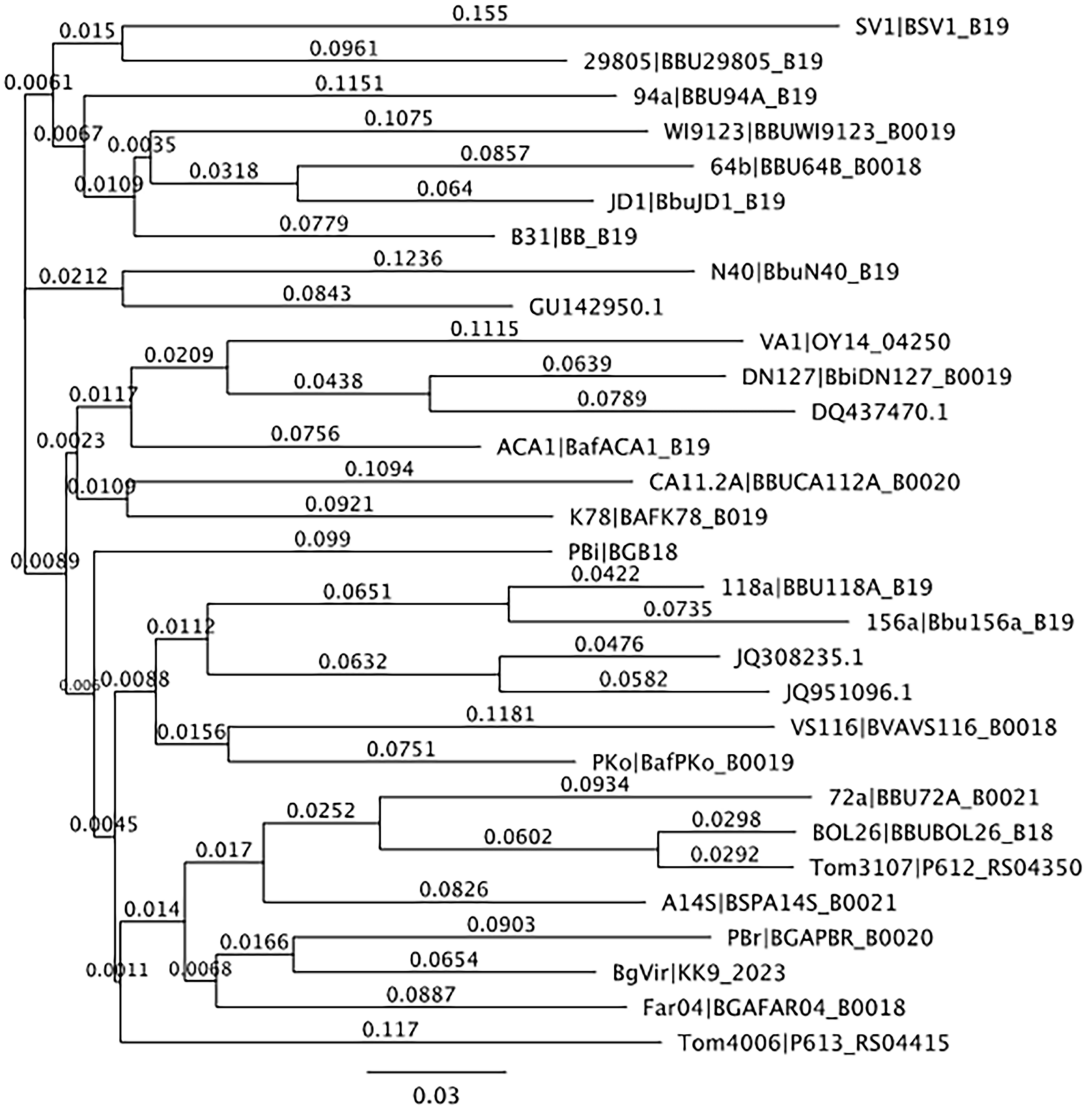
Neighbor-joining tree of reference sequences mapped to samples from this study. Neighbor-joining tree of OspC references observed in samples from this study created using Geneious version 10.0 created by Biomatters. Available from http://www.geneious.com.

From the 537 tissues sampled from 90 mice, a total of 163 tissues (30.4%) tested positive for *B. burgdorferi* infections. When mapped to the *Borreliella* types in the reference library at a 97% MOI, 143,894 sample sequences mapped to 10 of the original 30 reference sequences in the reference library. The frequency and distribution of these tissues to the mapped reference sequences was tabulated (Table 2) and visualized as phylogenetic trees (Figures 1, 2).

**Table 2:** *Borreliella burgdorferi* types in mouse tissue.

	*Borreliella burgdorferi sensu stricto*											Average # of types per tissue
	Accession #	DQ437444	DQ437449.1	CP001422	DQ437456	U01894.1	DQ437470.1	GU142950.1	DQ437462	JQ308235.1	JQ951096.1	
	Strain	118a	156a	64b	72a	B31	CS8		JD1	HPS61		
	Type	J	H2	B1	G	A	N		C1	I3		
*Tissue type*												
Ear		1	2	5	6	28	6	13	0	5	13	2.03
Heart		0	0	0	0	3	0	2	0	0	0	1
Kidney		0	0	0	0	4	0	0	0	0	0	1
Liver		0	1	2	1	22	4	5	0	2	2	1.39
Spleen		0	0	0	1	17	0	4	1	3	6	1.28
Tail		0	1	1	1	20	5	8	0	2	5	1.48
Tongue		0	3	3	3	25	8	9	1	2	5	1.79
TOTAL		1	7	11	12	119	23	41	2	14	31	

In terms of *Borreliella* variation, 143,894 of the initial 322,480 reads mapped to 10 of the reference sequences in the *ospC* strain library constructed for this study at a 97% MOI (Table 2). Two tissue samples, the ear and the tongue, each accounted for 90% of the observed *Borreliella* sequence diversity and combined they account for all of the variation seen in the tissue samples surveyed. The largest amount of variation observed in an individual tissue came from an ear tissue sample with six *ospC* sequence types that alone accounted for 60% of the variation observed across tested specimens. The breakdown of *B. burgdorferi* genetic types coinfecting individual tissue samples can be found in Table 3. Overall, the average number of types per infected tissue was the highest in the ear tissue with an average of 2.03 types observed. The remaining average number of types per infection by tissue was 1.79 per tongue, 1.48 per tail, 1.39 per liver, 1.28 per spleen, 1.0 per heart, and 1.0 per kidney.

**Table 3:** *Borreliella burgdorferi* number of types in individual tissues.

Number of tissues	Number of types present in one tissue sample
1	6
4	5
5	4
12	3
38	2
103	1

A statistically significant association was observed between tissue and the type of the *Borreliella* infection detected within those tissues. *Borreliella burgdorferi* type A (strain 72a) and JQ951096.1 were 3.58 and 2.94 times more likely to infect ear tissue than other tissue types (Tables 4, 5). The low frequency and narrow distribution of types found in all sampled tissues (spleen, liver, ear, tongue, tail, heart, and kidney) was not diversified enough across all sampled tissues to have statistical significance outside of type A (strain 72a) and JQ951096.1 in the ear. The Graves trapping location however had a 75% lower likelihood of trapping an infected mouse than other trapping locations (95% CI OR: 0.08, 0.71), which was statistically significant (value of *p* < 0.01).

**Table 4:** Statistical comparison of *Borreliella* type G (strain 72a) using logistic regression.

	Odds Ratio	STD ERR	Z-score	*p*-value	95% Conf. Int. OR	
Ear	3.58	2.181	2.09	0.037	1.08	11.82
Heart	1.00	(omitted)	predicts failure (Type G = 1 perfectly)			
Kidney	1.00	(omitted)	predicts failure (Type G = 1 perfectly)			
Liver	0.42	0.445	−0.82	0.413	0.05	3.37
Spleen	0.48	0.514	−0.69	0.493	0.06	3.90
Tail	0.40	0.425	−0.86	0.389	0.05	3.22
Tongue	1.34	0.937	0.42	0.671	0.34	5.27

**Table 5:** Statistical comparison of *Borreliella* type JQ951096.1 using logistic regression.

	Odds Ratio	STD ERR	Z-score	*P*-value	95% Conf. Int. OR	
Ear	2.94	1.251	2.54	0.011	1.28	6.77
Heart	1.00	(omitted)	predicts failure (JQ951096.1 = 1 perfectly)			
Kidney	1.00	(omitted)	predicts failure (JQ951096.1 = 1 perfectly)			
Liver	0.28	0.215	−1.66	0.096	0.06	1.25
Spleen	1.43	0.739	0.69	0.492	0.52	3.94
Tail	0.87	0.466	−0.27	0.788	0.30	2.48
Tongue	0.71	0.381	−0.63	0.528	0.25	2.03

## Discussion

Overall, 10 distinct *Borreliella* types identified from the 537 tissues tested in this study are all endemic to North America and were mostly described from the northeastern states of New York and Massachusetts. The observed diversity of these types was different between trapping locations and between tissue types. Interestingly, several tissues tested positive for more than one type, which should be studied further in the future in terms of immune response and competition between types. Prior studies established the correlation between OspC types and the invasiveness of infections seen in humans, specifically OspC types A, B, C, D, H, K, and N (Seinost et al., 1999; Earnhart et al., 2005; Lagal et al., 2006). The average number of types per infected tissue was also the highest in the ear tissue with an average of 2.03 types observed, which may be an evolutionary trait of *B. burgdorferi*, given that ticks will commonly attach around the ears of white-footed mice. The highest amount of variation observed came from the ear tissue of one mouse from the Transfer Station trapping location, which contained six different *Borreliella* types, representing 60% of the overall diversity found across all 537 tissue samples tested. This aligns with previous work that observed higher levels of *Borreliella* genetic diversity in isolates from skin tissue compared to blood, synovial, and cerebrospinal fluid from Lyme disease patients (Brisson et al., 2011).

The observed diversity of *ospC* in mouse tissues varied based on the trapping locations. Fairfax Transfer Station samples accounted for only 17.8% of the sample size but 100% of the observed diversity which can most likely be attributed to the consolidation of waste from around the county at this site. Overall, infection rates do not seem to be correlated with the amount of diversity in each site as the Goodwood site had a 66.7% infection rate, averaged the largest number of tissues infected per mouse at four, but had only two OspC types detected in positive tissue samples. Fairfax Transfer Station had an infection rate similar to Goodwood (68.8%), had fewer tissues infected per mouse (2), but had all 10 types observed for the entire study. It is important to note that the discrepancy between the total number of mice collected at each site was not evenly distributed, due to trap success rate. This lack of even distribution precluded statistical analysis of infection rate with respect to trapping location. The frequency distribution of the types was less evenly distributed than overall number of infected mice collected from trapping sites and therefore statistical significance of types was not reported.

A high degree of variability in the *ospC* of *B. burgdorferi* and its pervasive spread through all trapping locations surveyed in Fairfax County suggests that *Borreliella* is actively transmitted and maintained within white-footed mice populations. Fragmentated and highly disturbed habitats, such as those in Fairfax County, Virginia, naturally lend themselves to increased non-mouse reservoir population and further increase the risk of creating infected tick vectors (Brisson and Dykhuizen, 2004). Public health agencies should consider incorporating epigenetic surveillance into ongoing Lyme surveillance in reservoir hosts, collected ticks, and infected patients.

While legislation regarding testing and patient information has been in place for several years, clinicians are facing the challenge of increasing incidence of Lyme disease across the Northeast, with cases often presenting with vague symptoms beyond the telltale bullseye rash (The ILADS Working Group, 2004).

Many of the examined mice tested positive for more than one *Borreliella* type and several individual tissues tested positive for more than one type. The single tissue with the largest amount of diversity was an ear sample that contained six different *Borreliella* types, a total of 60% of the overall diversity found in all 537 tissue samples tested, which might serve as the optimal tissue sample for non-invasive genetic monitoring of *Borreliella* in white-footed mice (Zawada et al., 2020).

## Conclusion

The genetic diversity of *ospC* in the tongue and the ear samples each contained 90% of the overall observed sample diversity. Infections seen in the spleen, liver, ear, tongue, and tail tissues were all highly predictive of concurrent infections in other tissues from the same mouse at a 95% level of confidence. This suggests that in these five tissues, *Borreliella burgdorferi* infections are systemic rather than localized. Two OspC types were found to be statistically associated with being detected in a specific tissue, whereas type A (strain 72a) and JQ951096.1 were only found in ear tissue. To further explore the relationship between OspC type and tissue specificity, a larger sample size of white-footed mice would be required. Regardless, pairing genetic testing and surveillance of *Borreliella* in mammalian hosts with active cases of Lyme disease in neighboring populations, may hold important insights to drivers of tissue specific infection and the overall clinical management of acute and chronic Lyme disease infection.

## Data availability statement

All datasets presented in this study are included in the article/ Supplementary material.

## Ethics statement

The animal study was reviewed and approved by George Mason University, Institutional Biosafety Committee.

## Author contributions

SZ: conceptualization, methodology, formal analysis, lab analysis, sequencing, sample collection, investigation, and writing—original draft. MvF: methodology formal analysis, data visualization, and writing—original draft. TW: formal analysis, data visualization, and writing—editing and review. MS: methodology, lab analysis, sequencing, and writing—editing and review. PG: supervision, methodology, data curation, data visualization, and writing—editing and review. All authors contributed to the article and approved the submitted version.

## Funding

This project was supported using funds provided by George Mason University, Microbiome Analysis Center. Funding to cover publication costs was provided by George Mason University Libraries Open Access Publishing Fund.

## Conflict of interest

The authors declare that the research was conducted in the absence of any commercial or financial relationships that could be construed as a potential conflict of interest.

## Publisher’s note

All claims expressed in this article are solely those of the authors and do not necessarily represent those of their affiliated organizations, or those of the publisher, the editors and the reviewers. Any product that may be evaluated in this article, or claim that may be made by its manufacturer, is not guaranteed or endorsed by the publisher.

## Footnotes

^1^ http://borreliabase.org
